# Rapid Structural and Compositional Change in an Old-Growth Subtropical Forest: Using Plant Traits to Identify Probable Drivers

**DOI:** 10.1371/journal.pone.0073546

**Published:** 2013-09-17

**Authors:** Agustina Malizia, Tomás A. Easdale, H. Ricardo Grau

**Affiliations:** 1 CONICET-Instituto de Ecología Regional (IER), Universidad Nacional de Tucumán, Tucumán, Argentina; 2 Landcare Research, Lincoln, New Zealand; Centre National de la Recherche Scientifique, France

## Abstract

Recent studies have shown directional changes in old-growth tropical forests, but changes are complex and diverse, and their drivers unclear. Here, we report rapid net structural and compositional changes in an old-growth subtropical forest and we assess the functional nature of these changes to test hypothetical drivers including recovery from past disturbances, reduction in ungulate browsing, CO_2_ fertilization, and increases in rainfall and temperature. The study relies on 15 years of demographic monitoring within 8 ha of subtropical montane forest in Argentina. Between 1992 and 2007, stem density markedly increased by 50% (12 stems ha^−1^ y^−1^) and basal area by 6% (0.13 m^2^ ha^−1^ y^−1^). Increased stem density resulted from enhanced recruitment of understory treelets (*Piper tucumanum*, *Eugenia uniflora*, *Allophylus edulis*) into small size classes. Among 27 common tree species, net population growth was negatively correlated with maximum tree size and longevity, and positively correlated with leaf size and leaf nutrient content, especially so when initial population size was controlled for. Changes were inconsistent with predictions derived from past disturbances (no increase in shade-tolerant or long-lived late-succesional species), rainfall or temperature increase (no increase in evergreen or deciduous species, respectively). However, the increase in nutrient-rich soft-leaved species was consistent with exclusion of large herbivores two decades before monitoring started; and CO_2_ fertilization could help explain the disproportionate increase in small stems. Reductions in populations of large vertebrates have been observed in many otherwise undisturbed tropical forests, and our results suggest they can have important structural and functional repercussions in these forests.

## Introduction

The long-held assumption that old-growth forests are in long-term dynamic and structural steady state is currently under question for tropical forests, with indications that they are experiencing directional changes, e.g. Laurance et al. [1], Feeley et al. [2], Lewis et al. [3]. Analyses combining large monitoring plots from the American, African and Asian tropics indicate a widespread acceleration in tree recruitment and mortality between the 1980s and early 2000s [3]. Net increases in biomass and stem density resulted from increased rates of growth and recruitment that exceeded the increases in mortality. Across neotropical forests, stand basal area increased in 34 out of 50 plots monitored between 1985 and 1999 [4].

Potential explanations for the changing dynamics and biomass increase in tropical forests include: (a) long-term recovery from unrecorded past disturbances, (b) reduced ungulate herbivory, mainly due to hunting pressure, or changes in environmental drivers including: (c) increasing air temperatures (but see Clark et al. [5]), (d) rising atmospheric CO_2_ concentrations, (e) changes in rainfall, and (f) nutrient deposition from industrial and agricultural sources, see extensive reviews by Lewis et al. [6] and [7]. Unravelling the main drivers of change is difficult for several reasons, including the paucity of environmental and historical records in most forest locations, short observation windows, temporal co-variation among potential drivers, and delayed or non-linear responses [8]. Thus, the relative contribution of these potential factors is still uncertain, but dissecting the responses among tree diameter classes, habitats, and the demography and plant attributes of species with changing populations should contribute to falsify hypotheses and narrow down on potential drivers, e.g. Valencia et al. [9].

For example, one of the hypothesized drivers of change indicated above is that long-term changes in tropical forests may be due to the overhunting of large herbivores (i.e., >1 kg), which leads to the so-called ‘empty forests’ [10], [11]. Additionally, some forests have been experiencing reduced pressure by non-native herbivores (e.g. livestock) due to the abandonment of marginal productive land uses [12]. Because herbivores are selective in their consumption of seeds and seedlings [13], [14]; their reduced pressure is likely to preferentially release some species from herbivory and lead to compositional and structural changes [6], [15]. Thus, if old growth forests were experiencing a consistent proliferation of palatable species, this would signal loss of herbivory as a potential driver of change.

Thus far, the attention and debate about the direction, magnitude and, especially, the principal drivers of change in old-growth forests has primarily focused on tropical forests, particularly in the Neotropics, see Wright [7]. Together, the studies from Amazonia and Africa suggest that the average increase in forest-stand biomass is the outcome of concurrent increases of species with many different ecological habits, see Lewis et al. [3] and Text S1. Thus, there is a need to further understand the nature and extent of current changes. More broadly, if we want to anticipate how ecosystems will change in the future, it is important that we understand how species, traits and life histories respond to the increasingly disturbed and eutrophic landscapes of the 21^st^ century [16]. This is the motivation for our study within a subtropical forest.

Long-term monitoring of a subtropical montane forest in San Javier, NW Argentina, revealed that, between 1991 and 2006, old-growth forests have experienced a steep increase in turnover, and to a lesser extent an increase in basal area and biomass [17]. This region is subject to environmental changes comparable with those in many other forests – including increasing temperature and CO_2_, and decreased herbivory, both by native ungulates and livestock; however, unlike the decreasing rainfall registered in several tropical regions [3], this subtropical region has experienced increasing rainfall during recent decades [18], [19]. Demographic analyses have detected three orthogonal axes of life history variation for common tree species [20]: (1) A light-demand and growth-potential axis showed that species with faster diameter growth rates have more exposed crowns and lower abundance in old-growth forests compared with species with slower growth. (2) A population turnover (r-K) axis revealed that species with low rates of tree survivorship and short-lived individuals have, in old-growth forest, higher rates of recruitment, lower basal area, and size-class distributions with steeply negative slopes (few large individuals), compared with species that have high tree survivorship and long-lived individuals. (3) A third axis indicated that species that recruit commonly on landslides have low recruitment in old-field successions, suggesting different substrate requirements for establishment. Contrasts between plant traits measured on the same species at the same geographic location and the three demographic axes described above have shown that species with attributes typical of high capture and low conservation of resources – i.e., large and thin leaf laminas, high leaf K, P and N concentrations, light wood and small seeds – have faster population turnover than species with opposite attributes [21]. A link was also found between plant physiognomy and light demand: species with compound leaves, high ratio of leaf to stem mass, and tall first branch as saplings are more light demanding than species with opposite attributes [21].

In this study, we first examine structural and compositional changes over 15 years in an old-growth forest in the subtropical Andes. Then, we test probable drivers of change by examining what demographic life histories and morpho-physiologies are correlated with the observed changes. To this purpose, we relied on previous demographic analyses [20] and plant trait measurements [21] for common tree species. We tested the following causal hypothesis and attempted to formulate predictions that would help discriminate among hypotheses (Table 1) according to the following rationale:

**Table 1:** Summary of the hypothesis tested in this study.

Hypothesis	Prediction	Results of this study
Past disturbance (i.e., forests aresuccessional)	Increase in shade tolerant species with low growth potential (i.e. species on thenegative side of the *light demand and growth potential* axis), and increase inlong-lived species (i.e. species with slow *population turnover*)	No support for this prediction. Changes in forests structure are primarily not a result of recovering from past disturbances.
Decreasing herbivory	Increase in soft-leaved species with high nutrient concentrations in their leaves(i.e. species on the positive side of the *resource capture and conservation* axis)	Support for this hypothesis. Forest may by changing as a consequence of reduction of both native ungulates and livestock.
CO_2_ fertilization	Increase in understory trees (i.e. most stems in the smallest sizes)	Some effect. CO_2_ fertilization could partially contribute the observed changes.
Increasing rainfall	Increase in evergreen species (i.e. increase in species with higher*leaf evergreenness*)	No support for this prediction. Rainfall not being a limiting resource, may be influencing forests only to a minor extent.
Increasingtemperature	Increase in deciduous species and decrease in evergreen species	No support for this prediction. Maybe effects of more rainfall and more temperature compensate each other.
Population size	Positive correlation between population size and population change	Support for this predicion.

1. *Past disturbances.* If forests are recovering from past natural or anthropic large scale disturbance, we expect an increasing representation of slow-growing shade-tolerant species, and of long-lived late-successional species; which should be replacing the fast-growing and short-lived species that characterize early post-disturbance stages [22], [23]. In terms of structural adjustments, we expect a decrease in density of stems, particularly small-sized stems, and an increase in basal area, with concentrated growth in large trees [24].
2. *Herbivory reduction.* If forests are changing due to a reduction in herbivory (both by native ungulates or livestock), we expect an increase in density of stems of species with leaves that are ‘soft’ (e.g. higher specific leaf area) and have high nutrient concentration. This is because ‘soft-leaved’ species are usually more palatable than ‘tough-leaved’ counterparts with high fiber content [25]. This expectation is supported by empirical findings that exclusion of ungulates leads to increased recruitment of tree species with high nutrient content and low structural carbohydrate content [26].
3. *CO_2_ fertilization.* It has been suggested that high CO_2_ concentrations produce effects similar to illuminating the understorey [27]. Increased CO_2_ concentrations are known to enhance light-use efficiency [28] and various lines of evidence indicate that plant photosynthetic responses are more pronounced under low light, see reviews by Lewis et al. [6], and Körner [27]. This is expected to increase shade tolerance (i.e. survivorship under shade) for individual plants and, in turn, the above-ground biomass that can be packed in the forest understory. Thus, if forests are responding to increased atmospheric CO_2_ concentrations, we expect an increase in stem density and basal area of understory trees.
4. *Rainfall increase.* Tree ring studies conducted in a few dominant species have detected clear growth signals to the increased rainfall in the second half of the 20^th^ century in the seasonally-dry forests of NW Argentina [29], but the number of species assessed is insufficient to infer differential responses among them. Long-term monitoring has revealed increased mortality of evergreen species in response to exceptional droughts in Panamá [30] or reduced representation of evergreen species associated to decreasing rainfall in dry forests of Costa Rica [31] and in wet and moist forests in Ghana [32]. We would then interpret an increased representation of evergreen species as indication that the seasonally dry forest is responding to increasing rainfall.
5. *Temperature increase.* If increases in temperature are driving the observed structural changes, we expect an increase in basal area of deciduous species. Evergreen species typically possess conservative traits that limit nutrient loss (e.g. tissues with long lifespan and with low nutrient concentration). These are favourable plant attributes when nutrients are in short supply [33] and/or when environmental conditions such as ambient temperature limit growth e.g. van Ommen Kloeke et al. [34], but they limit a plant’s potential to maximize productivity when abundant resources and/or suitable environmental conditions favour growth. This expectation is supported by a recent meta-analyses of 63 studies (both experimental and field-based), which found that elevated temperatures favoured growth (height, stem diameter and biomass) in deciduous species more than in evergreen trees [35].
6. *Population size.* As growth conditions and essential resources vary, the vital rates of a species (e.g. individual growth, probability of mortality, number of seeds produced) will tend to track and adjust to the new conditions on a per capita basis [36]. Other things being equal (i.e. for functionally equivalent species), we expect that absolute rates of population change will be proportional to initial population size because all adjusted demographic rates will apply over a larger initial population. In other words, when conditions become more favourable and/or essential resources increase in availability (e.g. as ecosystems become eutrophicated), we expect that species with larger numbers of stems and basal area will experience greater *absolute* increases. Similarly, as conditions or resources become unfavourable, species with large populations will be those to experience greater *absolute* reductions.

## Materials and Methods

### Study Area

The study was conducted in Sierra de San Javier (26°45′ S, 65°19′ W), Tucumán, Argentina. The vegetation is typical of the lower elevation belt of the southern extension of Neotropical montane forests [37]. Old-growth forests are mostly located in steep topography and have an average of 23 tree species ha^−1^≥10 cm in diameter, including deciduous and evergreen species. The upper canopy layer (20–30 m high) is dominated by *Blepharocalyx salicifolius* (Myrtaceae), *Cinnamomum porphyrium* (Lauraceae) and *Pisonia zapallo* (Nyctaginaceae). The subcanopy (5–12 m high) is dominated by *Eugenia uniflora* (Myrtaceae), *Piper tucumanum* (Piperaceae) and *Allophylus edulis* (Sapindaceae). The understory consists of a relatively uniform layer of the shrub *Psychotria carthaginensis* (Rubiaceae). Light selective logging occurred ca. 60 years ago, probably for *Cedrela lilloi* (Meliaceae) and *Juglans australis* (Juglandaceae), with only two cut stumps found in the plot. The disturbance regime is dominated by treefall gaps [38], [39]. The forests were subject to extensive livestock browsing (mainly cattle and horses) for many decades up to 1973 when 14,000 ha of the Sierra were assigned to a protected area, which led to livestock exclusion, and socio-economic changes favored abandonment of marginal productive land uses [40]. Among the native herbivores, *Mazama gouazoubira*, a small deer, and *Sylvilagus brasiliensis*, a forest rabbit, forage on tree seedlings and saplings; and collared peccaries, *Pecari tajacu*, consume roots, seeds and fruits [41]. The area was subject to a process of severe defaunation in the past due to bushmeat poaching. For example, tapirs, *Tapirus terrestris*, were still present in the first half of the 20^th^ century but went locally extinct in 1940 [41]; and large herbivores decreased substantially in population density. In summary, herbivory pressure first changed from native fauna to livestock, then experienced an overall reduction approximately 30–40 years ago. More recently, the native fauna appears to show signs of slow recovery.

**Figure 1:**
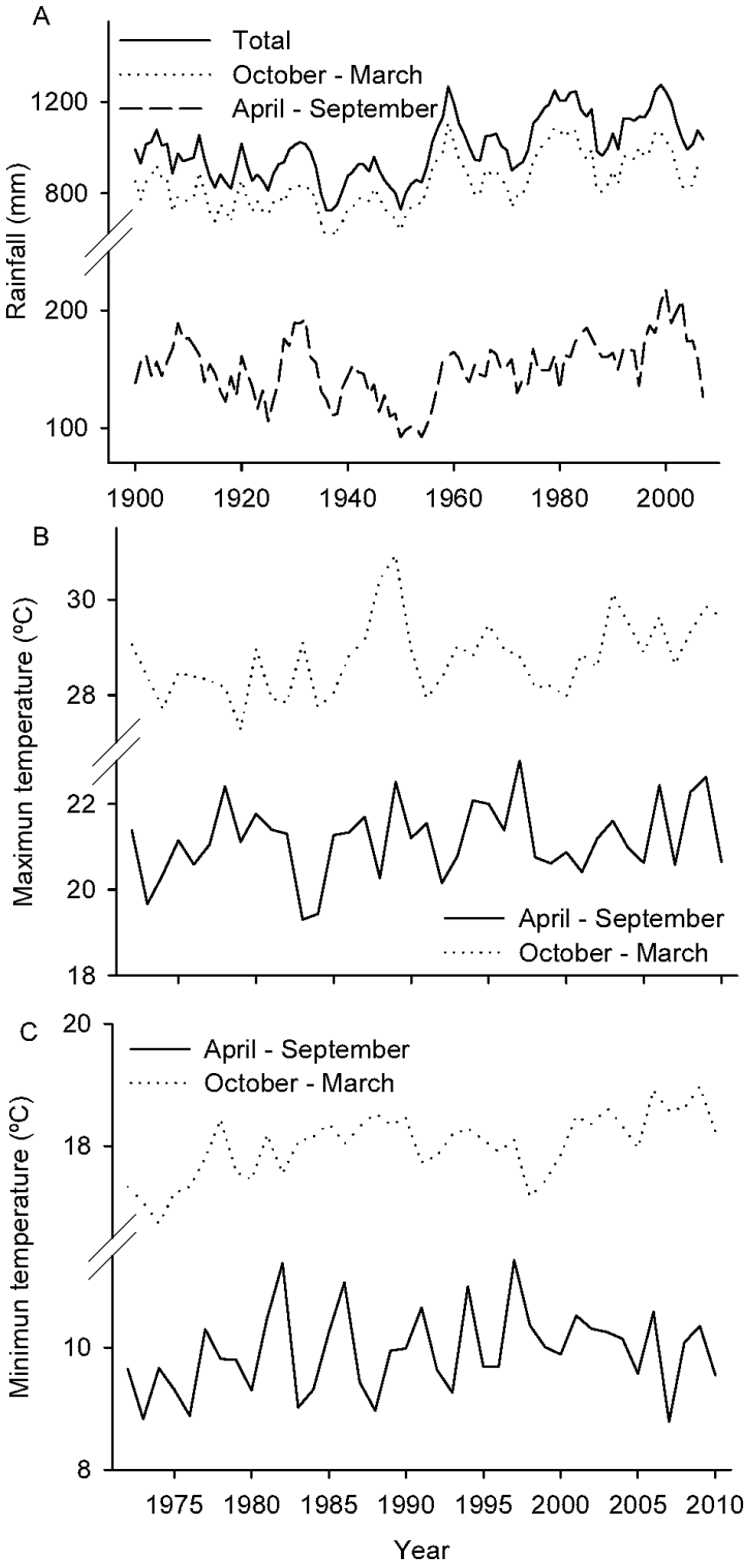
Rainfall and temperature records registered at Obispo Colombres Meteorological Station, Tucumán. a) Five year moving averages of annual rainfall (solid line), rainy season rainfall (October – March, dotted line) and dry season rainfall (April – September, dashed line) from 1900 to 2010; b) annual average of maximum and c) minimum temperatures during October – March (summer, dotted line), and April – September (winter, solid line) from 1972 to 2010. Rainfall and temperature data were registered at Obispo Colombres Meteorological Station, Tucumán (ca.500 m lower and 15 km east of the study site).

Annual rainfall ranges from 1300 to 1500 mm, and is distributed in a monsoonal regime with dry winters and wet summers [42]. The rainy season lasts from October to March and concentrates 80–90% of annual rainfall; the dry season extends from April to September. Mean annual temperature is 18°C, with frosts from June to August. Records from the nearest meteorological station (c. 500 m lower elevation and 15 km East of the study site) for the period 1900–2010 reveal a trend of increasing total annual rainfall between 1930 and 2000 with a stepwise increase in the late 1950s [18] and apparent stabilization since 2000 [19] (1950–2000: R^2^adj = 0.32, *P*<0.001, *b* = 5.17) (Fig.1a). This trend is the outcome of increasing rainfall during both the rainy (R^2^adj = 0.23, *P*<0.001, *b* = 3.81) and dry seasons (R^2^adj = 0.55, *P*<0.001, *b* = 1.36) (Fig. 1a). Temperature records from the same station for the period 1972–2010 show an increase in average maximum (R^2^adj = 0.16, *P*<0.01, *b* = 0.03) and minimum (R^2^adj = 0.37, *P*<0.001, *b* = 0.03) temperatures during October–March (rainy season) (Fig. 1b, c). It is difficult to assess to what extent the temperature increases are a result of urban expansion (cities tend to have higher air temperature than their rural surroundings) [43]; and if this phenomenon is affecting the study area.

The study is based on 15 years of records for 8-ha of permanent sample plots (one 6-ha plot of 200 × 300 m plus two nearby 1-ha plots) at ca. 1000 m asl [17]. The 6-ha plot was established in old-growth forest in1992 and the 1-ha plots were established and remeasured a year earlier, but we refer to the 1992-year for the 8-ha from hereafter. All trees ≥10 cm in diameter at breast height (dbh) were marked with aluminum tags nailed at breast height, identified to species, and measured (or recorded as dead) four times in 5-year intervals between 1992 and 2007. This study focuses on the 27 most common species, which accounted for 99.2% of the total basal area and 98.8% of the individuals (2095 in the first census).

### Data Analysis

To assess forest structural changes between 1992 and 2007, we computed the density of individual trees, the density of stems, and basal area for each measurement date (1992, 1997, 2002, 2007) both for the whole community and for each species. Then, we fitted linear regressions of each structural variable vs. time (with four data points each). The slope of each regression (*b*) was interpreted as a rate of change for each structural variable. The minimum absolute population change that could be detected was one new individual in 15 years within 8-ha (i.e. 0.0083 individual ha^−1 ^y^−1^).

To assess whether rates of structural change relate to particular life histories and/or plant traits, we ran partial Spearman rank correlations (controlling for initial population size) between the rate of structural change (*b*) and demographic variables and morphological traits measured on the same 27 common species. Demographic and morphological information was obtained from previous work conducted in the same sample plots and nearby secondary forests [20], [21]. Specifically, we tested both the individual demographic variables listed in Table S1 and species’ scores along three demographic PCA axes that summarize them (as described above, *light demand and growth potential*, *population turnover*, *substrate requirements for establishment*, Easdale et al. 2007). We also tested the plant traits listed in Table S2 and species’ scores along two morphological PCA axes summarizing them (*resource capture and conservation*, *compound-leaf physiognomy*) [21].

To test for a population-size effect, we contrasted standard Spearman correlations with partial Spearman rank correlations that controlled for a population-size effect (on the contrast between rates of structural change and species’ demographic and morphological traits). We favoured Spearman rank correlations over Pearson correlations to account for non-linear monotonic relationships. We had three reasons for (i) computing rates of change *b* and using partial correlations to account for the initial value of each structural variable (*Str*) instead of (ii) using a simple relative rate of change of the form (*Str_final_*−*Str_initial_*)/*Str_initial_*/(*Year_final_*−*Year_initial_*). First, we considered relevant to explicitly test for the size effect rather than hide it in a relative rate of change; second, the rate of change *b* provided a more robust estimate of change by accounting for all four measurements instead of just the first and last ones; third, by not having an intercept, a relative rate of change would incorrectly assume that structural change must be zero when the initial measurement is also zero (which is not necessarily the case with arrival of propagules from outside the plot or recruitment of small stems into 10 cm dbh).

Because our aim was to identify correlates and infer probable predictors of net population change for a common set of species growing at the same location, the study is necessarily correlational and this requires consideration when interpreting results. We note that although part of the data used to compute rates of structural change was also used to compute demographic rates, an association between them, if any, is not foreseeable. Net population change in any species results from imbalances between recruitment and mortality though various alternative mechanisms given by the initial size, direction, and rate of change of vital rates over time, see Fig. 1 in Lewis et al. [4]. For example, populations can increase in size even when their rates of mortality accelerate if they experience a greater proportional acceleration in recruitment rate.

## Results

### Temporal Changes at Stand-level

**Figure 2:**
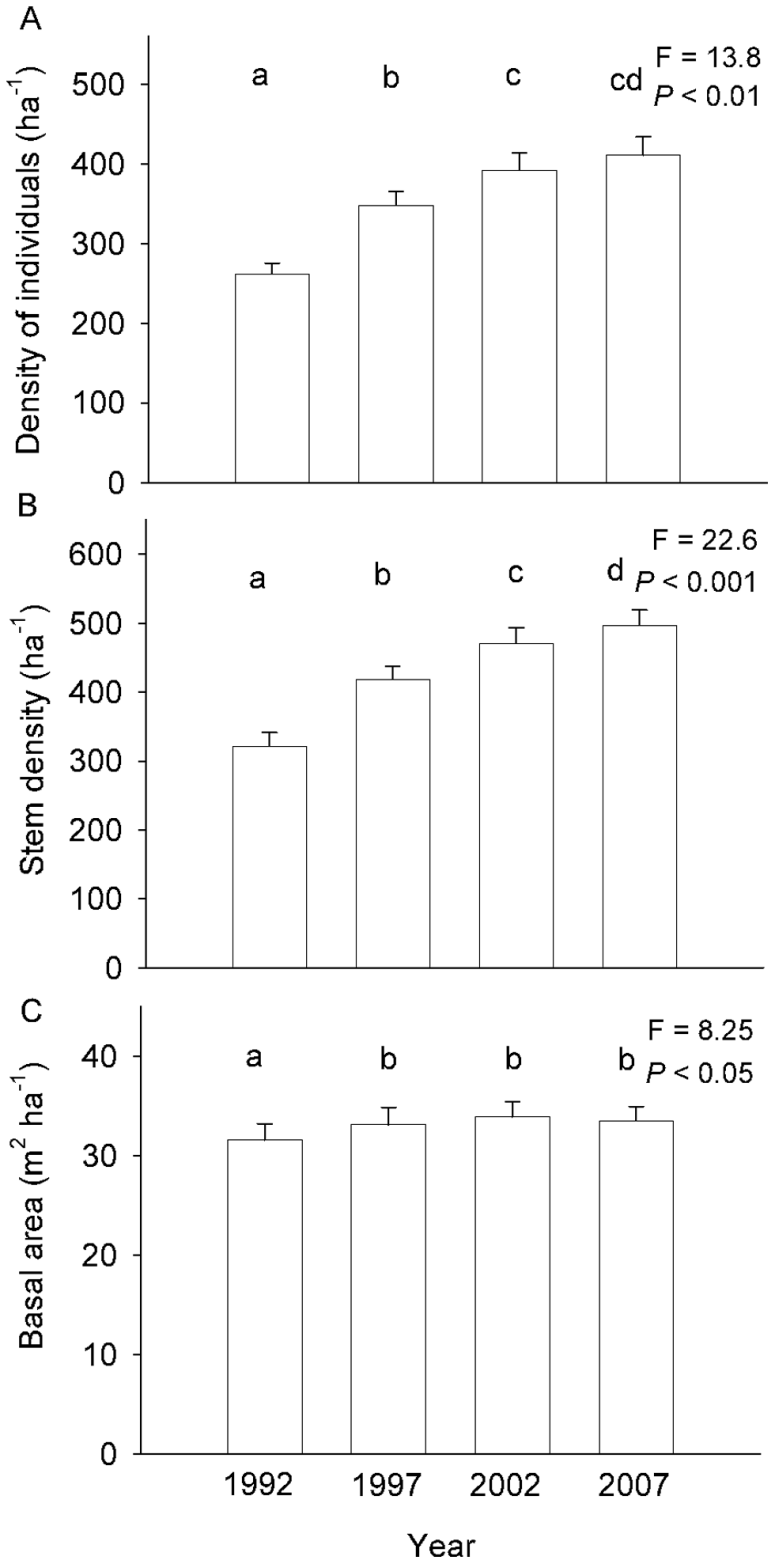
Structural changes in 15 years. a) Changes in density of individuals, b) density of stems, and c) basal area in all four census. Letters identify non-different groups from Friedman ANOVA and posteriori Wilcoxon match pair tests. Error bars correspond to standard error. All data are the per-hectare average of 8 ha.

**Figure 3:**
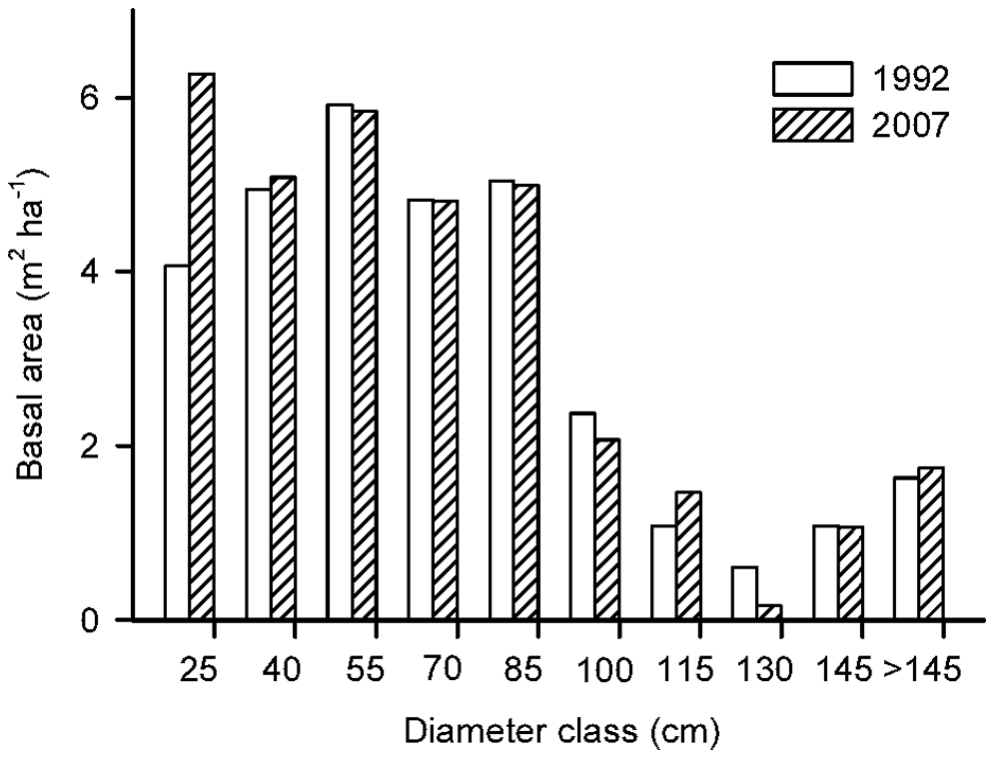
Basal area for 1992 and 2007 across 15(numbers indicate the upper limit of the class).

Between 1992 and 2007, there was a steep increase in both density of distinct individual trees (10 individuals ha^−1^ y^−1^), and density of stems (12 stems ha^−1^ y^−1^) (57 and 55%, respectively) (Fig. 2a, b), and a comparatively minor (6%) increase in basal area (0.13 m^2^ ha^−1^ y^−1^) (Fig. 2c). The increase in density was spatially consistent, occurring in each one of the 8 hectares and was largely concentrated in stems between 10 and 25 cm dbh, which increased by more than 30% of their initial basal area (Fig. 3). The 115–130 cm dbh size class experienced a basal area reduction of more than 50% (Fig. 3), but this was due to the death of a single individual of *C. porphyrium* while other size classes remained relatively stable.

### Temporal Changes at Species-level

**Figure 4:**
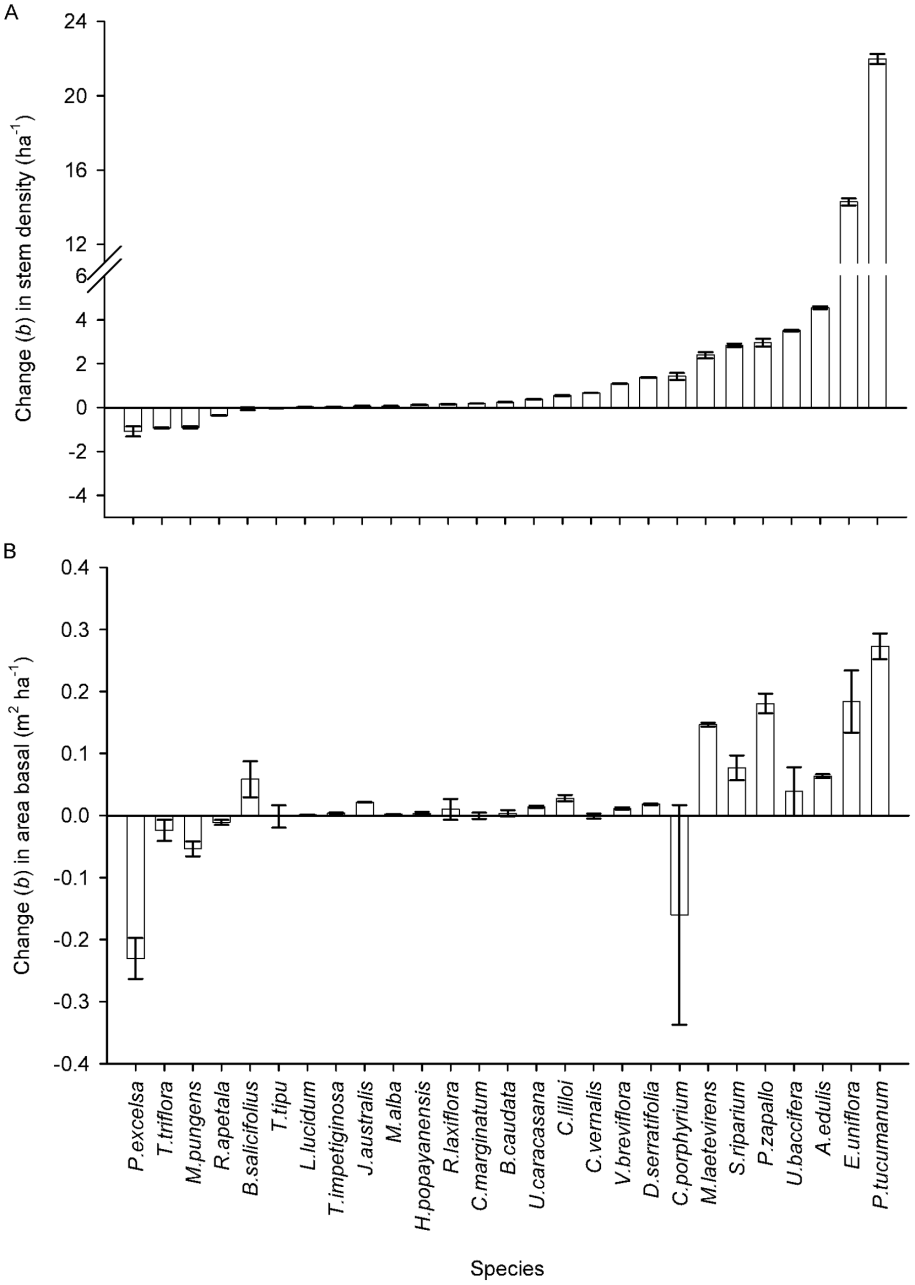
Changes in stem density and basal area across species in 15 years. Slope of the regression (rate of change) calculated for a) stem density, and b) basal area for each species across 15 years. Error bars correspond to standard error.

Among species, temporal changes in stem density ranged from −1.09 to 21.99 stems ha^−1 ^yr^−1^ (Fig. 4a). Two subcanopy species (*E. uniflora* and *P. tucumanum*) showed major positive changes in stem density, followed by 11 other species that also increased, while 10 species showed minor or no change. Only four species (the subcanopy species *M. pungens* and *R. apetala* and the canopy species *T. triflora* and *P. excelsa*) decreased in stem density within the recorded period (Fig. 4a), but these species have relatively low densities and their impact on total stem density change was comparatively minor. The same trend was found for density of individuals (results not shown). Temporal changes in basal area ranged from −0.23 to 0.27 m^2^ ha^−1^ yr^−1^, with 14 species increasing, 8 species showing minor or no change and 5 species decreasing in basal area (the subcanopy *M. pungens*, and *R. apetala,* and the canopy species *T. triflora, C. porphyrium* and *P. exelsa*) (Fig. 4b). Among species, changes in stem density were positively correlated with changes in basal area (Spearman r = 0.73, p<0.001). Greater rates of population change (in absolute value) were recorded in species with larger populations in 1992, both for stem density (Spearman r = 0.60, p<0.001) and for basal area (Spearman r = 0.64, p<0.001).

### Correlates of Structural Change

Various demographic variables and plant traits were correlated with the structural changes (Tables 2 and 3). The relationship between demographic or morphological variables and structural changes (*b*) followed similar patterns when using standard or partial Spearman rank correlations that controlled for initial population size; but partial correlations tended to be stronger and more significant than standard correlations (Tables 2 and 3). When initial population size was controlled for, greater net increases in density of stems, individuals, and basal area were found in species with high rates of recuitment (to ≥10 cm dbh) and steep negative exponential size distributions in old-growth forest. Also, greater net increases in density of stems and individuals were found for species with low basal area in old-growth forest, and short-lived trees that only attain small dbh and stature (Table 2). Assessments with plant traits showed that the greater net increases (*b*) in density of stems and individuals were found in species with large and thin leaf laminas, high leaf K and P concentrations, and light wood (when initial population size was controlled for). Changes in basal area were positively correlated with specific leaf area and leaf K content (Table 3).

**Table 2:** Table 2. Standard Spearman rank correlations and partial Spearman rank correlations (that control for initial population sizes) between the rate of structural change (*b*) and demographic variables measured on the 27 most common species at San Javier.

Demographic Axes	Demographic variable	n	*b* basal area	*b* basal area | initial basal area	*b* stems	*b* stems | initial stems	*b* individuals	*b* individuals | initial individuals
*Light demand and growth* *potential* (PC1)	Maximum growth rate Co	27	−0.12	−0.13	−0.14	−0.05	−0.15	−0.08
	Basal area SF	27	−0.22	−0.22	−0.21	−0.18	−0.22	−0.19
	Mean growth rate under well-lit conditions Co	27	−0.05	−0.06	−0.04	0.05	−0.05	0.03
	Mean growth rate under shade conditions OGF	24	−0.16	−0.17	0.02	0.08	0.00	0.05
	Crown illumination index Co	27	−0.2	−0.21	−0.07	0.02	−0.07	0.00
	Stem density SF	27	−0.11	−0.11	−0.12	−0.11	−0.13	−0.12
	Stem density OGF	27	0.35	**0.53** **	**0.48** *	NA	**0.46** *	**0.81** ***
	Shade tolerance index Co	27	0.17	0.19	0.28	0.21	0.26	0.20
*Population turnover* (PC2)	Relative recruitment rate OGF	24	**0.51** *	**0.52** *	**0.73** ***	**0.81** ***	**0.75** ***	**0.81** ***
	Minimum tree longevity Co	27	−0.31	−0.39	−0.35	**−0.59** **	−0.38	**−0.59** **
	Slope OGF	24	**−0.55** **	**−0.56** **	**−0.70** ***	**−0.73** ***	**−0.73** ***	**−0.75** ***
	Basal area OGF	27	−0.01	NA	−0.05	**−0.45** *	−0.08	**−0.43** *
	Relative mortality rate Co	27	−0.04	−0.06	0.13	0.19	0.16	0.21
	Slope SF	19	−0.11	−0.11	−0.30	−0.28	−0.33	−0.31
*Substrate requirements for establishment* (PC3)	Extreme pioneer index	27	−0.2	−0.24	−0.26	−0.18	−0.23	−0.17
	Growth rate variability SF	20	0.01	0.03	−0.02	−0.16	−0.02	−0.15
	Relative recruitment rate SF	21	0.28	0.28	0.40	0.36	0.39	0.35
*Variables not related to any demographic axes*	Mean growth rate under shade conditions SF	21	0.04	0.04	0.01	0.07	−0.02	0.03
	Growth rate variability OGF	23	−0.27	−0.26	−0.26	−0.29	−0.25	−0.27
	Maximum unified dbh	27	−0.32	−0.36	**−0.43** *	**−0.49** *	**−0.46** *	**−0.51** **
	Maximum height	27	−0.24	−0.24	**−0.46** *	**−0.46** *	**−0.48** *	**−0.48** *
	Number of stems per individuals	27	−0.2	−0.20	0.08	0.02	0.06	0.02

SF: Secondary Forest, OGF: Old Growth Forest, Co: combined SF and OGF datasets. Bold numbers indicate significant correlations.

* *P*<0.05,

** *P*<0.01,

*** *P*<0.001.

**Table 3:** Standard Spearman rank correlations and partial Spearman rank correlations (that control for initial population sizes) between the rate of structural change (*b*) and morphological variables measured on the 27 most common species at San Javier.

Morphological axes	Plant traits	n	*b* basal area	*b* basal area| initial basal area	*b* stems	*b* stems| initial stems	*b* individuals	*b* individuals| initial individuals
*Resource capture and* *conservation* (PC1)	Leaf lamina size	27	0.32	0.35	**0.41** *	**0.53** **	**0.41** *	**0.52** **
	Leaf K	27	**0.41** *	**0.43** *	**0.57** **	**0.66** ***	**0.57** **	**0.65** ***
	Specific leaf area	27	**0.55** **	**0.57** **	**0.55** **	**0.62** ***	**0.56** **	**0.62** ***
	Leaf P	27	0.04	0.03	**0.40** *	**0.47** *	**0.40** *	**0.47** *
	Leaf N	27	0.18	0.18	0.37	0.37	0.37	0.38
	Wood density	27	−0.31	−0.31	**−0.47** *	**−0.55** **	**−0.46** *	**−0.53** **
	Seed mass	27	−0.22	−0.22	−0.16	−0.21	−0.19	−0.23
*Physiognomic features* (PC2)	Leaf type	27	−0.15	−0.15	−0.2	−0.16	−0.2	−0.17
	Leaf/above-ground weight ratio	25	−0.04	−0.05	0.1	0.16	0.08	0.13
	First branch height_125_	25	0.14	0.14	0.16	0.26	0.18	0.27
*Variables not related to any morphological axes*	Leaf evergreenness	27	0.01	0.01	0.26	0.21	0.24	0.20
	Leaf Ca	27	0.15	0.14	−0.09	−0.04	−0.08	−0.03
	Crown width_125_	25	−0.07	−0.07	0.04	−0.05	0.00	−0.08

SF: Secondary Forest, OGF: Old Growth Forest, Co: combined SF and OGF datasets. Bold numbers indicate significant correlations.

* *P*<0.05,

** *P*<0.01,

*** *P*<0.001.

**Figure 5:**
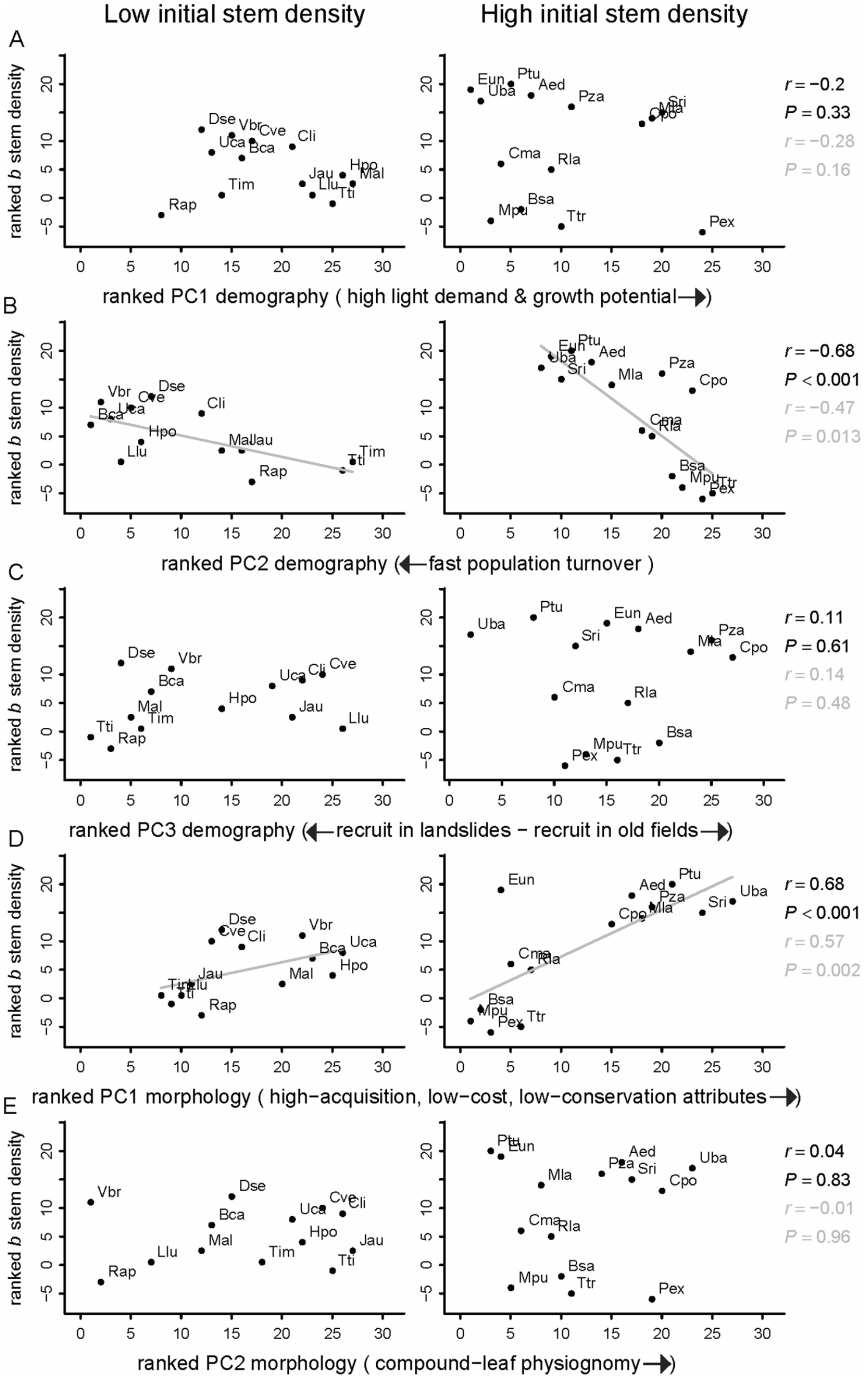
Relationship between stem density changes in 15 years and species life histories and morphological traits. Coplots between the trend of change (*b*) in stem density and demographic and morphological principal components (PC), for low (left) and high (right) initial stem density in 1992. Plotted values are ranks. To aid interpretation, ranked axes were rescaled so that negative *b* in stem density matched with negative ranks. Correlations in black correspond to partial Spearman correlations; values in grey represent standard Spearman correlations (that did not control for initial stem density). Regression lines are for graphical reference only. See Table S3 for species code abbrevattions.

The above results for individual demographic variables and plant traits become more clear when we assess the results for demographic and morphological principal components that summarize species’ life histories and functional ‘strategies’. We found a negative partial correlation between the rate of change (*b*) in stem density and *population turnover* (demographic PC2); species with fast rates of turnover showed greater absolute increases in stem density, but the increase was steeper for species that had larger populations in 1992 (Fig. 5b). The largest increases in stem density were observed in short-lived species with high mortality rates, size distributions with a steep negative slope, low basal area, and high rates of recruitment (to ≥10 cm dbh.) in old-growth forest (*P. tucumanum*, *E. uniflora*, *A. edulis*, *Urera baccifera*, *Solanum riparium* and *M. laetevirens*) although two species without such traits also increased their abundance (*P. zapallo* and *C. porphyrium*). We also detected a positive partial correlation between the rate of change (*b*) in stem density and plant attributes characteristic *of high capture and low conservation of resources* (morphological PC1), which was more notable in species with high initial stem density (Fig. 5d). Species that increased in stem density tend to have large and thin leaf laminas, high leaf K, P and N content, and (with the exception of *E. uniflora*) low wood density and small seeds.

**Figure 6:**
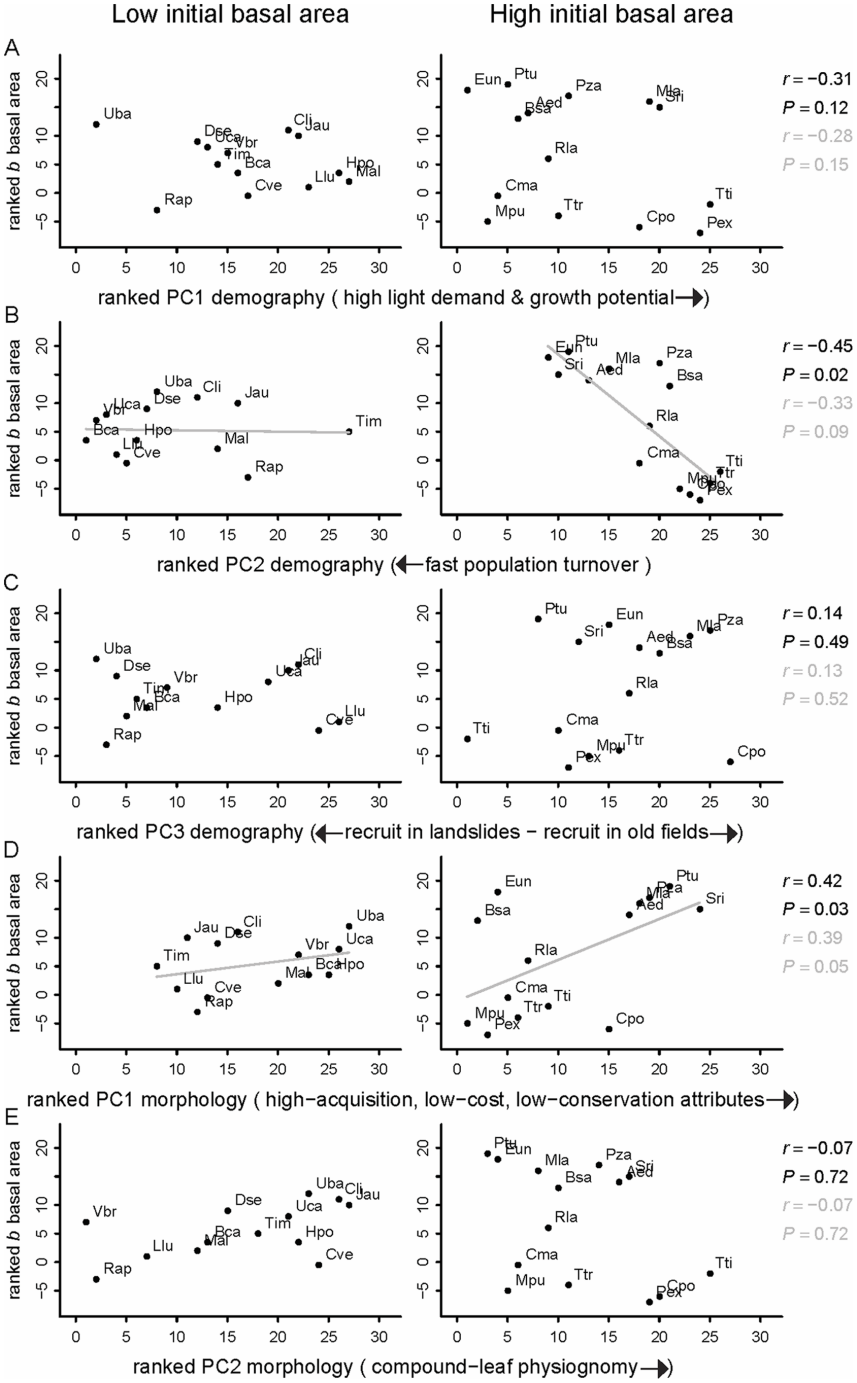
Relationship between area basal changes in 15 years and species life histories and morphological traits. Coplots between the trend of change (*b*) in basal area and demographic and morphological principal components (PC), for low (left) and high (right) initial basal area in 1992. Plotted values are ranks. To aid interpretation, ranked axes were rescaled so that negative *b* in basal area matched with negative ranks. Correlations in black correspond to partial Spearman correlations; values in grey represent standard Spearman correlations (that did not control for initial basal area). Regression lines are for graphical reference only. See Table S3 for species code abbrevattions.

Structural changes in basal area followed the same patterns as those in stem density. We found a negative partial correlation between the rate of change (*b*) in basal area and *population turnover*, which was steeper for species with high initial basal area (Fig. 6b). The species with the largest increases in basal area (*P. tucumanum, E. uniflora, S. riparium, M. laetevirens* and *A. edulis*) tend to be short-lived species, with high population turnover in old-growth forest. We also found a positive partial correlation between the rate of change in basal area and plant traits characteristic *of high capture and low conservation of resources*, mainly for species with high initial basal area (Fig. 6d).

## Discussion

### Temporal Changes at Stand-level

The above results reveal that this old-growth subtropical montane forest experienced consistent and rapid changes in forest structure between 1992 and 2007. The most salient feature of these changes was a steep increase (55%) in density of stems by an average of 12 stems ha^−1^ y^−1^, mainly those between 10 and 25 cm dbh. This was due to the net addition both of stems in multi-stemmed trees and distinct individual trees. The net increase in stem density resulted in a comparatively smaller increase (6%) in total basal area of 0.13 m^2^ ha^−1^ y^−1^. It is important to note here that the major increase in density of small stems and main cause of structural change, cannot be attributed to a methodological artefact of tags being nailed at breast hight.

Our recorded increase in total basal area of 0.13 m^2^ ha^−1^ y^−1^ is very similar to a net increase of 0.10±0.04 m^2^ ha^−1^ y^−1^ reported over a 30-year period (1971–2002) for 50 plots distributed across the South American tropics [4]. In contrast, the steep net increase in stem density of 12 stems ha^−1^ y^−1^ recorded for this forest is an order of magnitude higher than the mean increase of 0.94±0.63 stems ha^−1^ y^−1^ reported by Lewis et al. [4] and to a later estimate of 0.73±0.61 stems ha^−1^ y^−1^ weighted by sampling effort across 95 plots in the same study area [3]. While in Amazonia the net addition of small stems only amounted to ca. 7% of stand-level basal area growth, much of the structural change recorded in this subtropical forest is precisely attributed to increased density of small stems, which accounted for ca. 72% of stand-level growth in basal area.

### Changes at Species-level and Drivers of Change

Changes in stem density and basal area were not homogeneous among species. When initial population size was accounted for, the greater increases both in stem density and basal area were detected in species with *fast population turnover* in old-growth forest (short-statured short-lived stems due to high rates of mortality that are compensated for by high recruitment rates) and with functional attributes typical of *high acquisition of resources* (i.e. large and thin leaf laminas, high leaf K and P concentrations, and light wood). These compositional changes differ from previous studies in Amazonia [3], where the average increase in forest stand biomass was caused by increases in species with a wide range of ecological habits. Our results indicate that increases in stem density were different from zero in 17 species whereas decreases were different from zero in only 4 species. This suggests that one or more resource or growing condition that is limiting to most species has increased. What does this mean in terms of the potential causes of change introduced above?

#### Disturbance

There was no association between rates of structural change and species shade tolerance and growth potential, thus indicating that the changes were not the result of post-disturbance succession. There was no evidence of an increased presence of shade tolerants as expected towards later successional stages nor an increased presence of light demanders as expected with increased fine-scale disturbance (e.g. treefall gaps). We did find a clear increase of short-lived species over long-lived late-successional species but this was not spatially associated to canopy gaps resulting from the death of one or a few clustered trees, and must be attributed to a different cause. We found no sign of the accumulation in basal area of large-sized trees that is expected when stands progress towards later successional stages [24], [44]. Moreover, although this is a dynamic forest with regular formation of gaps [39], the basal area (an estimate of canopy cover) of large canopy trees and of intermediate subcanopy trees (which also release essential resources and favour small trees and saplings; Connell et al. [45]) remained stable. It thus seems unlikely that the most important changes in this old-growth forest are the result of a post-disturbance successional process. Some canopy species that do recruit in recently disturbed areas (e.g. *Parapiptadenia excelsa*) do not show recruitment in the understory and are gradually decreasing their canopy density, as one would expect in post-disturbance stands, but this is not a significant process when analyzed at the whole-community scale.

#### Herbivory/livestock

‘Soft-leaved’ species with large and thin leaf laminas, and high leaf K and P content tend to be more palatable than species with contrasting attributes [25]. The steep population increase observed for species with functional attributes typical of *high acquisition of resources* is consistent with expectations of change due to the defaunation that took place during the 19^th^ and 20^th^ centuries [41] and the more recent exclusion of livestock when the area became a protected area, as a part of broader socio-economic and management changes [40]. While having a limited impact in terms of leaf area consumed, herbivores may have marked effects on plant communities through their selective grazing and browsing [13], and their absence may release some species leading to functional shifts in communities and ecosystems [46]. In turn, these shifts may lead to major changes in ecosystem processes giving rise to indirect effects via trophic cascades or physical habitat modification [47]. Thus, the observed changes may have consequences at ecosystem level that deserve future study.

#### CO_2_ concentrations

One well documented physiological response of plants to elevated CO_2_ is an improvement in light-use efficiency. This appears to impact on the ecological performance of shaded plants and is expected to enhance the density of stems in the understory [6], [27]. The relative effect of CO_2_ increase on tropical plants grown in deep shade can be dramatic and, according to some interpretations, it can even exceed any effect seen under optimal horticultural conditions in full light [27]. The trends observed in this study appear to be consistent with this prediction, since there was a clear increase in density of stems in the smallest recorded size class (which were also likely to be more shaded).

#### Rainfall

Total annual rainfall increased in the second half of the 20^th^ century in northwestern Argentina [18]. Although some evergreen species such as *P. tucumanum* and *E. uniflora* showed an increase in stem density and basal area, leaf evergreenness as a whole did not correlate with any structural change. Contrary to findings reported for some regions in Central America [30], [31] and in Ghana [32] where forests show shifts from a more mesic to a more xeric composition in response to decreasing water availability, in our study case, a relatively large rainfall increase does not appear to have a major effect on the relative abundance of tree species.

#### Temperature

Although the average minimum and maximum temperatures for the rainy season have increased by 0.3°C during the growing-season each decade over the last 40 years c. 500 m below our study site, the largest basal area or population increases were not necessarily concentrated on deciduous species; giving no support for the hypothesis that increasing temperatures are responsible for the changes recorded. The compositional changes expected from this hypothesis are opposite to those expected from increasing rainfall and it seems possible that the effects of more or less simultaneous increases in rainfall and temperature may have compensated each other. It is worth noting that, throughout the monitoring period, the rising temperatures have not impacted negatively in this subtropical montane forest. Warming experiments with saplings of five temperate tree species have shown a distinct potential of physiological acclimation and suggests that the direct impact of climatic warming on tree growth and survival may *per se* be less critical than anticipated, at least at intermediate latitudes and altitudes [48]. The temperate climate is characterized by greater seasonal and day-to-day variation in temperature [49] compared to moist tropical forests, e.g. Clark et al. [5] and there is now supporting evidence that, compared with tropical species, temperate species have a greater capacity to adjust to fluctuations in temperature [50]; and that they can maintain maximum net photosynthesis over a wider span of growth temperatures [49].

#### Population size

As the availability of limiting resources or the suitability of environmental conditions change in time, trees shall adjust their vital rates to those new conditions. This study provides support for the expectation that locally abundant species will show greater absolute rates of structural change compared with less abundant species. In our interpretation, the basic mechanism mediating this response is that adjusted demographic rates and probabilities apply to a larger initial population and so a larger number of individuals will contribute to population growth or decline, see Silvertown and Charlesworth [36]. The alternative that growing conditions (e.g. climate) may have been more favourable for recruitment in some species through a storage effect [51] cannot be completely discarded but seems unlikely in that the recruitment pulse was mostly restricted to dominant species. Admittedly, the size effect is not exempt from sampling artifacts. Because for a given effect size (i.e. the degree of change to detect), the statistical power (the probability of correctly rejecting a null hypothesis when it is false) increases as a function of sample size [52], we cannot discard the possibility that less abundant or dominant species are also changing. But the practical implication of our results is not trivial: common and dominant species will likely show the greater and faster responses to changes in resource availability or environmental conditions. Does the relatively low tree diversity and greater abundance of common species in this subtropical forest explain the greater net increases in stem density compared with those reported by Lewis et al. [3] for the Amazon? Are old-growth temperate and boreal forests being more responsive to environmental change? These seem challenging but relevant directions for future research.

## Conclusions

An old-growth subtropical forest is experiencing relatively rapid structural, functional and compositional shifts. Species with high initial density of stems and individuals, and to a lesser extent basal area, are making a stronger contribution to those changes. Building on previous studies in tropical forests [1], [3], [53], [54] we found that species’ rates of change were linked to their demographic life histories and functional traits. Short lived and statured species with *fast population turnover* and attributes characteristic of *high resource acquisition* (e.g. soft nutrient-rich leaves) show a greater rate of change. A reduction in ungulate herbivory, a relatively ubiquitous phenomenon, and CO_2_ fertilization appear to be the most likely drivers of change while population size emerges as an important mediating variable. These findings emphasised that the role of altered biotic interactions needs to be considered as a possible driver of change in forests beside climate drivers. A greater understanding of the life history and functional ecology of individual species in different settings should help to further understand how forests are responding to global change.

## Supporting Information

Table S1 Summary of demographic variables computed at San Javier (see Easdale et al. (2007) for details). SF: Secondary Forest, O-GF: Old-Growth Forest, Co: combined SF and OGF datasets. Loadings represent the importance of each variable on each “demographic” PCA axis, the absolute number represents the magnitude, and the sign indicates the direction of the association.
(DOC)

Table S2 Summary of plant morphological traits measured previously at San Javier (see Easdale & Healey (2009) for details). The subscript 125 stands for a 125cm-tall sapling. Loadings represent the importance of each variable on each “morphological” PCA axis, the absolute number represents the magnitude, and the sign represents the direction of association.
(DOC)

Table S3 Common tree species registered at San Javier, Argentina. Nomenclature follows Zuloaga & Morrone (1999). Family names follow the Angiosperm Phylogeny Group (2003).
(DOC)

Text S1 Studies in the tropics that have investigated population changes over 20–25 years in tree species with different demography and plant attributes.
(DOC)
