# Supplementary material for: Rapid Structural and Compositional Change in an Old-Growth Subtropical Forest: Using Plant Traits to Identify Probable Drivers
Source: PLoS One. 2013 Sep 17;8(9):e73546. doi: 10.1371/journal.pone.0073546 (PMC3775741; doi:10.1371/journal.pone.0073546)
Supplement: Table S1 — Summary of demographic variables computed at San Javier (see Easdale et al. (2007) for details). SF: Secondary Forest, O-GF: Old-Growth Forest, Co: combined SF and OGF datasets. Loadings represent the importance of each variable on each “demographic” PCA axis, the absolute number represents the magnitude, and the sign indicates the direction of the association. (DOC) [file pone.0073546.s001.doc]

**Rapid structural and compositional change in an old-growth subtropical forest: using plant traits to identify probable drivers**

Agustina Malizia^1*^, Tomas A. Easdale^2^, H. Ricardo Grau^1^

*Corresponding author: e-mail: [agustinamalizia@yahoo.com](mailto:agustinamalizia@yahoo.com)

**Table S1.** Summary of demographic variables computed at San Javier, Argentina (see Easdale et al. (2007) for details). SF: Secondary Forest, O-GF: Old-Growth Forest, Co: combined SF and OGF datasets. Loadings represent the importance of each variable on each “demographic” PCA axis, the absolute number represents the magnitude, and the sign indicates the direction of the association.

| **Demographic axes** | **Demographic variables** | **Loadings** | **Description** |
| --- | --- | --- | --- |
| *Light demand and growth potential* (PC1) | Maximum growth rate Co | 0.86 | 90th percentile (i.e. upper 10%) of 5-year tree diameter growth rates for stems 10-40 cm dbh |
|  | Basal area SF | 0.81 | Stem (≥10 cm dbh) cross-sectional area per ground area |
|  | Mean growth rate under well-lit conditions Co | 0.81 | Mean diameter growth rates of trees 10-40 cm dbh with ≥ 50% of their crowns exposed to direct vertical light |
|  | Mean growth rate under shade O-GF | 0.73 | Mean diameter growth rates of trees 10-40 cm dbh with < 50% of their crowns exposed to direct vertical light |
|  | Crown illumination index Co | 0.7 | Mean crown illumination index (eight categories: 1, 1.5, 2, 2.5, 3, 3.5, 4 and 5) for trees 10-30 cm dbh; 1 are completely overshadowed treelets whereas 5 are emergent trees. |
|  | Density of individuals SF | 0.68 | Computed with individuals (≥10 cm dbh) |
|  | Density of individuals O-GF | -0.66 | Computed with individuals (≥10 cm dbh) |
|  | Shade tolerance index Co | -0.63 | Density of saplings (0.3–1.5m tall) growing in the shade (i.e. crown illumination index ≤2.5) divided by density of trees (≥10 cm dbh) within the three subplots (20 x 20m) with the highest density of trees for each species |
| *Population turnover* (PC2) | Relative recruitment rate O-GF | -0.85 | Number of tree (≥10 cm dbh) recruits in 5 years divided by the number of living individuals ≥10 cm dbh at the beginning of the period (potential seeders) |
|  | Minimum tree longevity Co | 0.81 | The species minimum expected time to reach the maximum measured dbh from 10 cm dbh was estimated with maximum diameter growth curves (90th percentile of growth rate) computed with a boot-strapping technique |
|  | Slope O-GF | 0.76 | The slope of a linear regression between ln(number of individuals per size class + 1) and the midpoint of each size class. The diameter range of each species (10 cm to the 95th percentile of individual dbh) was divided into eight equal size classes to control for interspecific differences in maximum size. |
|  | Basal area O-GF | 0.66 | Stem (≥10 cm dbh) cross-sectional area per ground area |
|  | Relative mortality rate Co | -0.57 | Number of tree (≥10 cm dbh) which died within 5years divided by the number of living individuals ≥10 cm dbh at the start of the period |
|  | Slope SF | 0.45 | Same as “slope O-GF” but in SF |
| *Substrate requirements for establishment* (PC3) | Extreme pioneer index | -0.7 | Indicates capacity to: (1) recruit under shade only (non-pioneer), (2)recruit both under shade and on landslides or not found in either circumstance (intermediate), and (3) recruit on landslides only (extreme pioneer) |
|  | Growth rate variability SF | -0.64 | *cv* of diameter growth rate of trees 10-40 cm dbh |
|  | Relative recruitment rate SF | 0.61 | Same as Relative recruitment rate O-GF but in SF |
