# Supplementary material for: Rapid Structural and Compositional Change in an Old-Growth Subtropical Forest: Using Plant Traits to Identify Probable Drivers
Source: PLoS One. 2013 Sep 17;8(9):e73546. doi: 10.1371/journal.pone.0073546 (PMC3775741; doi:10.1371/journal.pone.0073546)
Supplement: Table S2 — Summary of plant morphological traits measured previously at San Javier (see Easdale & Healey (2009) for details). The subscript 125 stands for a 125cm-tall sapling. Loadings represent the importance of each variable on each “morphological” PCA axis, the absolute number represents the magnitude, and the sign represents the direction of association. (DOC) [file pone.0073546.s002.doc]

**Rapid structural and compositional change in an old-growth subtropical forest: using plant traits to identify probable drivers**

Agustina Malizia^1*^, Tomas A. Easdale^2^, H. Ricardo Grau^1^

*Corresponding author: e-mail: [agustinamalizia@yahoo.com](mailto:agustinamalizia@yahoo.com)

**Table S2.** Summary of plant morphological traits measured previously at San Javier, Argentina (see Easdale & Healey (2009) for details). The subscript 125 stands for a 125cm-tall sapling. Loadings represent the importance of each variable on each “morphological” PCA axis, the absolute number represents the magnitude, and the sign represents the direction of association.

| **Morphological axes** | **Plant traits** | **Loadings** | **Description** |
| --- | --- | --- | --- |
| *Resource capture and conservation* (PC1) | Leaf lamina size | 0.84 | Projected lamina area for simple leaves; mean leaflet area for compound leaves (mm^2^) |
|  | Leaf K | 0.81 | Measured with a flame emission photometer in ground leaf samples (mg g^-1^) |
|  | Specific leaf area | 0.78 | Projected leaf size divided by its dry weight (mm^2^ mg^-1^) |
|  | Leaf P | 0.71 | Measured by colorimetry in ground leaf samples (mg g^-1^) |
|  | Leaf N | 0.67 | Measured with a Leco CHN analyzer (mg g^-1^) |
|  | Wood density | -0.8 | Dry weight/fresh volume for two perpendicular increment cores sampled from stems 25-40 cm dbh (g cm^-3^) |
|  | Seedmass | -0.72 | Air-dried weight of mature, undamaged seeds without attachments (g) |
| *Physiognomic features*  (PC 2) | Leaf type | 0.79 | Treated as an ordinal variable: (1) simple leaves, (2) palmate compound leaves, and (3) pinnate and bi-pinnate compound leaves |
|  | Leaf/above-ground mass ratio | 0.74 | Ratio of the dry weight of leaves to leaves plus stems in harvested saplings between 53 and 224 cm height |
|  | First branch height_125_ | 0.59 | Height at which the lowest branch joins the main stem in saplings between 53 and 224 cm in height (cm) |
