# Supplementary material for: Rapid Structural and Compositional Change in an Old-Growth Subtropical Forest: Using Plant Traits to Identify Probable Drivers
Source: PLoS One. 2013 Sep 17;8(9):e73546. doi: 10.1371/journal.pone.0073546 (PMC3775741; doi:10.1371/journal.pone.0073546)
Supplement: Table S3 — Common tree species registered at San Javier, Argentina. Nomenclature follows Zuloaga & Morrone (1999). Family names follow the Angiosperm Phylogeny Group (2003). (DOC) [file pone.0073546.s003.doc]

**Rapid structural and compositional change in an old-growth subtropical forest: using plant traits to identify probable drivers**

Agustina Malizia^1*^, Tomas A. Easdale^2^, H. Ricardo Grau^1^

*Corresponding author: e-mail: [agustinamalizia@yahoo.com](mailto:agustinamalizia@yahoo.com)

**Table S3.** Common tree species registered at San Javier, Argentina. Nomenclature follows Zuloaga & Morrone (1999). Family names follow the Angiosperm Phylogeny Group (2003).

| **Plant Family** | **Species names** | **Code** |
| --- | --- | --- |
| Bignoniaceae | *Tabebuia impetiginosa* (Mart. ex DC.) Standl. | Tim |
| Combretaceae | *Terminalia triflora* (Griseb.) Lillo | Ttr |
| Fabaceae | *Parapiptadenia excelsa* (Griseb.) Burkart | Pex |
| Fabaceae | *Tipuana tipu* (Benth.) Kuntze | Tti |
| Juglandaceae | *Juglans australis* Griseb. | Jau |
| Lauraceae | *Cinnamomum porphyrium* (Griseb.) Kosterm. | Cpo |
| Malvaceae | *Heliocarpus popayanensis* Kunth | Hpo |
| Meliaceae | *Cedrela lilloi* C. DC. | Cli |
| Moraceae | *Morus alba* L. ***** | Mal |
| Myrsinaceae | *Myrsine laetevirens (Mez)* Arechav | Mla |
| Myrtaceae | *Eugenia uniflora L.* | Eun |
| Myrtaceae | *Myrcianthes pungens* (O. Berg) D. Legrand | Mpu |
| Myrtaceae | *Blepharocalyx salicifolius* (Kunth) O. Berg | Bsa |
| Nictaginaceae | *Pisonia zapallo* Griseb.var. *zapallo* | Pza |
| Oleaceae | *Ligustrum lucidum* W.T. Aiton ***** | Llu |
| Piperaceae | *Piper tucumanum* C. DC. | Ptu |
| Poligonaceae | *Ruprechtia laxiflora* Meisn. | Rla |
| Poligonaceae | *Ruprechtia apetala* Wedd. | Rap |
| Sapindaceae | *Allophylus edulis* (A. St.-Hil., Cambess. & A. Juss.) Radlk. | Aed |
| Sapindaceae | *Cupania vernalis* Cambess. | Cve |
| Sapotaceae | *Chrysophyllum marginatum* (Hook. & Arn.) Radlk. | Cma |
| Solanaceae | *Solanum riparium* Pers. | Sri |
| Solanaceae | *Vassobia breviflora* (Sendtn.) Hunz. | Vbr |
| Urticaceae | *Urera baccifera* (L.) Gaudich | Uba |
| Urticaceae | *Urera caracasana* (Jacq.) Gaudich ex Griseb. | Uca |
| Urticaceae | *Boehmeria caudata* Sw. | Bca |
| Verbenaceae | *Duranta serratifolia* (Griseb.) Kuntze | Dse |
