# Supplementary material for: Rapid Structural and Compositional Change in an Old-Growth Subtropical Forest: Using Plant Traits to Identify Probable Drivers
Source: PLoS One. 2013 Sep 17;8(9):e73546. doi: 10.1371/journal.pone.0073546 (PMC3775741; doi:10.1371/journal.pone.0073546)
Supplement: Text S1 — Studies in the tropics that have investigated population changes over 20–25 years in tree species with different demography and plant attributes. (DOC) [file pone.0073546.s004.doc]

**Rapid structural and compositional change in an old-growth subtropical forest: using plant traits to identify probable drivers**

Agustina Malizia^1*^, Tomas A. Easdale^2^, H. Ricardo Grau^1^

*Corresponding author: e-mail: [agustinamalizia@yahoo.com](mailto:agustinamalizia@yahoo.com)

**Text S1**. A limited number of studies in the tropics have investigated population changes over 20-25 years in tree species with different demography and plant attributes. Findings and interpretations vary among these studies. For example, Feeley et al. (2011) detected a temporal increase in species with heavy wood and tall stature in the seasonally humid forests of Barro Colorado island, Panama. In the tropical dry forests of Guanacaste, Costa Rica, Enquist and Enquist (2011) found an increase in the proportion of deciduous and compound-leaved canopy species. Both studies, conducted in locations with marked rainfall seasonality, interpreted the observed floristic changes as responses to increased drought. Although the latter is a successional forest, the observed floristic changes seem to diverge from expected successional trajectories to mature forest (Enquist and Enquist 2011). In old-growth rain forests of Amazonia, Laurance et al. (2004) found an increase in fast-growing tree genera, including many canopy and emergent species and suggested that rising atmospheric CO_2_ concentrations explained those changes. In a pantropical study, Chave et al. (2008) found an increase in biomass, particularly of the slowest-growing species, and in species with the smallest seed size, but no significant change in biomass in relation to wood density or maximum tree size, and they concluded that plots were recovering from unknown past disturbances. Lewis et al. (2009b) reported relative changes in species biomass across tropical African forests, but found no relationship between the wood density of species and their change in biomass, relative to the stand.
